# A predictive analytics model for differentiating between transient ischemic attacks (TIA) and its mimics

**DOI:** 10.1186/s12911-020-01154-6

**Published:** 2020-06-18

**Authors:** Alia Stanciu, Mihai Banciu, Alireza Sadighi, Kyle A. Marshall, Neil R. Holland, Vida Abedi, Ramin Zand

**Affiliations:** 1 Freeman College of Management, Bucknell University, 1 Dent Drive, Lewisburg, PA 17837-2005 USA; 2 Department of Neurology, Division of Cerebrovascular Diseases, Geisinger Medical Center, 100 N Academy Ave, Danville, PA 17822 USA; 3 Department of Emergency Medicine, Medicine Institute, Geisinger Medical Center, 100 N Academy Ave, Danville, PA 17822 USA; 4 Department of Molecular and Functional Genomics, Weis Center for Research, Geisinger Health System, 100 N Academy Ave, Danville, PA 17822 USA; 5 Biocomplexity Institute of Virginia Tech, 1015 Life Science Circle, Blacksburg, Virginia 24061 USA; 6 Geisinger Commonwealth School of Medicine, 525 Pine St., Scranton, PA 18509 USA

**Keywords:** Diagnostic error, TIA, Transient ischemic attack, Stroke, Stroke mimic, Feature selection, Classification, Machine learning, Prospective study, TIA clinic, Clinical decision support

## Abstract

**Background:**

Transient ischemic attack (TIA) is a brief episode of neurological dysfunction resulting from cerebral ischemia not associated with permanent cerebral infarction. TIA is associated with high diagnostic errors because of the subjective nature of findings and the lack of clinical and imaging biomarkers. The goal of this study was to design and evaluate a novel multinomial classification model, based on a combination of feature selection mechanisms coupled with logistic regression, to predict the likelihood of TIA, TIA mimics, and minor stroke.

**Methods:**

We conducted our modeling on consecutive patients who were evaluated in our health system with an initial diagnosis of TIA in a 9-month period. We established the final diagnoses after the clinical evaluation by independent verification from two stroke neurologists. We used Recursive Feature Elimination (RFE) and Least Absolute Shrinkage and Selection Operator (LASSO) for prediction modeling.

**Results:**

The RFE-based classifier correctly predicts 78% of the overall observations. In particular, the classifier correctly identifies 68% of the cases labeled as “TIA mimic” and 83% of the “TIA” discharge diagnosis. The LASSO classifier had an overall accuracy of 74%. Both the RFE and LASSO-based classifiers tied or outperformed the ABCD2 score and the Diagnosis of TIA (DOT) score. With respect to predicting TIA, the RFE-based classifier has 61.1% accuracy, the LASSO-based classifier has 79.5% accuracy, whereas the DOT score applied to the dataset yields an accuracy of 63.1%.

**Conclusion:**

The results of this pilot study indicate that a multinomial classification model, based on a combination of feature selection mechanisms coupled with logistic regression, can be used to effectively differentiate between TIA, TIA mimics, and minor stroke.

## Background

Transient ischemic attack (TIA) is defined as a brief episode of neurological dysfunction resulting from cerebral ischemia not associated with permanent cerebral infarction [1]. Diagnosis or suspicion of TIA has become essential in stroke prevention due to the higher risk of subsequent stroke among TIA patients [2]. However, due to the lack of clinical biomarkers and subjective nature of the findings in most patients, accurate diagnosis of TIA is challenging [3–5]. While TIA underdiagnosis can have significant consequences, studies have indicated a high rate of TIA overdiagnosis [6, 7] which can be a burden for healthcare systems [8]. Researchers have developed several clinical risk scores [9] for predicting recurrence following a cerebral ischemic episode, including the well-studied ABCD2 scoring system [10]. However, the reliability of these scoring system for differentiating between a TIA and its mimics is questionable [11–13].

There is an increasing body of medical literature that relies on advanced statistical tools for analysis, classification, and prediction of health care-derived data. Examples of such methodologies include both binomial and multinomial logit models, coupled with multivariate models among others. At the same time, there is also a growing need to refine these models to better understand and predict the key contributing factors and their associations with respect to the specific condition under investigation.

The goal of this study was to design and evaluate a novel multinomial classification model, based on a combination of feature selection mechanisms coupled with logistic regression, to predict the likelihood of TIA, TIA mimics, and minor stroke. Methods based on logistic regression approaches have been employed by several of the current scoring systems for TIA and stroke [14], to predict 30-day recurrence in either stroke or TIA [15]. Other various multivariate models have been successfully used to compute, among other outcomes, reliable risk scores for patients with [16] and without [17] atrial fibrillation (AF) admitted with acute ischemic stroke or TIA.

## Methods

### Patient population

We analyzed consecutive patients with TIA-like symptoms who presented to the emergency department in one of our three tertiary stroke centers or our single TIA clinic in central and northeast Pennsylvania during a 9-month period. TIA-like symptoms were defined as abrupt but transient (less than 24 h) a) hemisensory or hemimotor symptoms affecting the face, arm and leg, b) aphasia or dysarthria, c) visual defect, d) lack of awareness, and e) vertigo or loss of balance or coordination. Our system TIA guidelines mandate both the primary care and ED providers to refer all suspected TIA patients, regardless of their risk profile, for urgent inpatient or outpatient (same-day TIA clinic) evaluation. Patients with low ABCD2 score are usually referred to our same-day TIA clinic; however, patients with high risk profile or patients who present over weekends are admitted to the hospital. All hospitalized patients with an initial diagnosis of TIA were initially evaluated by an emergency department (ED) provider, to exclude other possible etiologies causing the symptoms (hypoglycemia, infection, significant electrolyte abnormalities), and subsequently by a neurologist within 24 h. Each patient had at least one hospital discharge follow-up visit with a board-certified neurologist or vascular neurologist within 3 months. For this study, we used the tissue based definition of TIA [1] that excludes patients with permanent cerebral infarction. Therefore, patients who did not have a brain MRI were excluded from the study, to eliminate a permanent cerebral infarction. Patients who did not have an outpatient follow-up visit were also excluded from this study. The Institutional Review Boards of Geisinger and Bucknell University approved this study; written informed consent was waived.

### Verification of diagnosis

In order to build the benchmark dataset, we validated each patient’s final diagnosis. We manually reviewed all patients’ baseline characteristics including demographics, vascular risk factors, clinical work-up, neuroimaging, as well as discharge diagnoses. We also carefully reviewed patients’ initial symptoms, the sequence of the events, duration of symptoms, the nature of symptoms (focal vs. non-focal), corresponding vascular territory, the anatomy of symptoms, associated symptoms, and other possible differential diagnoses. The final diagnosis was classified as either TIA, TIA mimics, or minor stroke. The TIA diagnosis category includes all the patients who had the diagnosis of TIA or probable TIA (where a cerebrovascular diagnosis was the most likely, but other diagnoses were considered as well). The minor stroke category contains patients who had a positive neuroimaging for acute stroke while their symptoms resolved within 24 h. Patients with TIA mimic had other diagnoses that were mimicking cerebral ischemia and resolved within 24 h (e.g., migraine headache, Todd’s paralysis, etc.).

The final diagnosis was made independent of the hospital discharge diagnosis. Among patients who had a hospital discharge follow-up visit outside of our stroke clinic, the final diagnosis was made by consensus between our stroke research fellow and one of our vascular neurologists, who reviewed the cases independently. For the remaining patients who were seen in our stroke clinic by one of our vascular neurologists, the final diagnosis was independently verified by our stroke research fellow based on all clinical information. In either situation, when there was not a consensus, a second vascular neurologist reviewed the case and acted as a tiebreaker.

### Predictive analytics model

#### Dataset preparation and sampling

We used 269 consecutive patients with a TIA initial diagnosis to develop our model. In order to address the relative imbalance among the classes, we performed data augmentation using the Synthetic Minority Over-sampling Technique (SMOTE) algorithm [18]. SMOTE generates synthetic data points via convex combinations of nearest-neighbors from each class. These additional points are added to each member class of the predictor in a controlled manner, such that under-represented classes are over-sampled and over-represented classes are under-sampled. In the end, each category in the predictor class had a combination of real and synthetic data points, with the proportion of points in each category being controlled exogenously. For our analysis, we chose to sample up to 300 observations, broken down in a 100:150:50 split across the three classes of interest so that the incidence of the phenotype with lower prevalence is still low and does not distort the relative original proportions; thus the “TIA mimics” class is under-sampled, whereas the “TIA” and “minor stroke” classes are over-sampled. This final data set was partitioned into a training set containing 70% of the data and a testing set comprising the remaining 30% of the data. We also standardized all continuous variables.

#### Feature selection

The second step of the analysis involved selecting an appropriate set of features that are relevant for the predictive procedure. The original data contains a mix of 62 clinical and demographical features, and while it is easy to include all of them in a predictive model, it is quite likely that not all of them may have predictive power. The data mining literature is rich regarding feature selection methods and we ultimately decided on implementing, for comparison purposes, two different feature selection methods: Recursive Feature Elimination (RFE) [19] and Least Absolute Shrinkage and Selection Operator (LASSO) [20, 21]. RFE recursively “prunes” features deemed not to be important for predicting the discharge diagnosis by optimizing at each step the training cost function and then assigning a rank to each feature that contributes to the objective. The feature with the lowest rank is eliminated and RFE runs again recursively on the smaller set of features. If there is an improvement in the objective function, a new feature is eliminated, otherwise, the procedure terminates. The final set of features is then passed to a multinomial logit classifier.

#### Model development

Recall that in a multinomial logistic model with *K* total features and where the predictor has *J* different categories, the probability of the *i*-th observation belonging to category *j*, 1 < *j* < *J*, is given by:

$$ {\displaystyle \begin{array}{l}\Pr \left({y}_i=j|\mathbf{x},\boldsymbol{\upbeta} \right)=\frac{e^{\sum \limits_{k=0}^K{x}_{ik}{\beta}_{kj}}}{1+{\sum}_{j=1}^{J-1}{e}^{\sum \limits_{k=0}^K{x}_{ik}{\beta}_{kj}}},\kern0.5em j<J\\ {}\Pr \left({y}_i=J|\mathbf{x},\boldsymbol{\upbeta} \right)=\frac{1}{1+{\sum}_{j=1}^{J-1}{e}^{\sum \limits_{k=0}^K{x}_{ik}{\beta}_{kj}}},\kern0.5em j=J,\end{array}} $$

where for each dependent categorical outcome *y*_*i*_ (the discharge diagnostic), *x*_*ik*_ is the *k*-th feature describing observation *i* and *β*_*kj*_ is the regression estimate for the *k*-th feature associated with outcome *j*. The coefficients are estimated using the maximum log-likelihood method, that is, the function

$$ L\left(\boldsymbol{\upbeta} \right)=\sum \limits_{i=1}^N\sum \limits_{j=1}^{J-1}\left({y}_i\sum \limits_{k=0}^K{x}_{ik}{\beta}_{kj}\right)-{n}_i\log \left(1+{\sum}_{j=1}^{J-1}{e}^{\sum \limits_{k=0}^K{x}_{ik}{\beta}_{kj}}\right) $$

is maximized using all *β*_*kj*_ as decision variables and with *n*_*i*_ denoting the proportion of items belonging to class *i* from the dataset. In contrast, the LASSO works by adding a penalty term to the log-likelihood function. The penalty term is the L1-norm of the coefficients so that during the minimization procedure there is a strong incentive to set the coefficients associated with the weak predictors to zero, i.e., eliminate the corresponding feature from the model. The adjusted (penalized) objective function thus becomes:

$$ {L}_p\left(\boldsymbol{\upbeta} \right)=L\left(\boldsymbol{\upbeta} \right)-\lambda \sum \limits_{k=0}^K\sum \limits_{j=1}^{J-1}\left|{\beta}_{kj}\right| $$

where *λ* is a regularization parameter (the magnitude of the penalty). Thus, in the LASSO model, feature selection happens simultaneously with the actual classification, as some of the parameters *β*_*kj*_ will be set to 0 in the optimal solution, to reduce the magnitude of the penalty. As we will see below, each of these feature selection mechanisms yields slightly different performances, with RFE being better at identifying TIA and the LASSO being more accurate at identifying both “TIA mimic” and “minor stroke” categories. In the LASSO model, because of the L1-regularization, the final set of roughly 30 features may be different across classes. We implemented all models in Python 3.7 using the libraries pandas [22], numpy, imbalanced-learn [23], and scikit-learn [24].

## Results

Out of 269 consecutive patients (mean age: 69.9 ± 15.1, 56.5% men) with an initial diagnosis of TIA, 50.2% had the final diagnosis of TIA. Table 1 presents the patients’ demographic and clinical information. The supplemental Table 1 displays the list of clinical and imaging elements considered for the diagnosis of TIA. The majority (71.3%) of the patients had a follow-up visit at our hospital-discharge stroke clinic; however, several patients (28.7%) had follow-up appointments in general neurology or primary care offices. The inter-rater agreement for the final diagnosis of TIA was 80.9% (κ = 0.62).

**Table 1:** Patient demographic information

**Total patients, no (%)**	269
Gender, Male, no (%)	152 (56.5%)
Age, Mean ± SD	69.9 ± 15.1
Median ABCD2 Score	4
**Race**	
White	261 (97.0%)
Black or African American	7 (2.6%)
Declined to Provide	1 (0.4%)
**Medical History**	
Hypertension	206 (76.6%)
Atrial Fibrillation	43 (16.0%)
Hyperlipidemia	214 (79.6%)
Seizure	12 (4.5%)
Headache (any type)	49 (18.2%)
Migraine without aura	19 (7.1%)
Migraine with aura	11 (4.1%)
Carotid Disease	163 (60.6%)
Anticoagulant Use	26 (10.0%)
Tobacco Use	61 (22.7%)
**Clinical Observations**	
Altered Mental Status^a^	51 (19.0%)
Aphasia	51 (19.0%)
Numbness	129 (48.0%)
Weakness	128 (47.6%)
Headache	59 (21.9%)
Dysarthria	87 (32.3%)
Facial Droop	52 (19.3%)
Sudden True Vertigo	11 (4.1%)
Diplopia	6 (2.2%)
Mono-ocular Blindness	4 (1.5%)
Hemianopsia	35 (13.0%)
Ataxia	35 (13.0%)
Seizure-like Activity	6 (2.2%)
Visual Aura	7 (2.6%)
Pre-syncope	43 (16.0%)
**Discharge Diagnostic Category**	
TIA mimics	103 (38.3%)
TIA	135 (50.2%)
Minor Stroke	31 (11.5%)

^a^Altered Mental Status was assessed based on level of consciousness (LOC), LOC Questions, and LOC Commands as defined in the National Institutes of Health Stroke Scale (NIHSS)

We first present the performance of the multinomial RFE-based classifier on the synthetic test set (generated using the SMOTE procedure). Table 2 includes the final list of predictors left in the multinomial logit model after applying RFE with a cutoff of 20 features and retaining only those features with p-values < 0.05 for at least one category, together with their associated coefficients, standard errors, corresponding odds ratios, and p-values.

**Table 2:** Diagnostic discharge predictors – RFE feature selection

	Coefficient (*β*_*i*_)	SE	Odds Ratio	*P*-value
**Discharge Diagnosis: TIA mimics**				
Altered mental Status (0,1)^a^	0.551	0.420	1.734	0.190
Hx of AF, PAF, A. Flutter (0,1)	−1.033	0.505	0.356	0.041
Hx of HTN (on Medication) (0,1)	1.395	0.384	4.034	0.000
Hx of Hyperlipidemia (on Medication) (0,1)	0.675	0.404	1.964	0.095
Hx of Seizure (0,1)	−1.657	0.811	0.191	0.041
Language disturbance-Expressive Aphasia (0,1)	−0.220	0.405	0.802	0.587
Numbness (Leg, Arm, or facial) (0,1)	0.109	0.356	1.115	0.759
Pre-TIA OAC (0,1) - Coumadin, Pradaxa, Eliquis (apixaban), Xarelto	0.618	0.629	1.855	0.326
Tobacco	0.766	0.406	2.152	0.059
Weakness (general, unilateral arm or leg) (0,1)	−0.840	0.328	0.432	0.010
Hx of Carotid Disease	−0.202	0.608	0.817	0.740
Intercept	−0.637	0.562	0.529	0.257
**Discharge Diagnosis: TIA**				
Altered mental Status (0,1)^a^	−1.494	0.698	0.225	0.032
Hx of AF, PAF, A. Flutter (0,1)	0.036	0.575	1.037	0.950
Hx of HTN (on Medication) (0,1)	1.509	0.540	4.522	0.005
Hx of Hyperlipidemia (on Medication) (0,1)	−0.726	0.483	0.484	0.133
Hx of Seizure (0,1)	−0.811	0.843	0.444	0.335
Language disturbance-Expressive Aphasia (0,1)	0.330	0.503	1.391	0.511
Numbness (Leg, Arm, or facial) (0,1)	−0.784	0.442	0.456	0.076
Pre-TIA OAC (0,1) - Coumadin, Pradaxa, Eliquis (apixaban), Xarelto	−0.072	0.771	0.931	0.926
Tobacco	−0.498	0.588	0.608	0.397
Weakness (general, unilateral arm or leg) (0,1)	0.443	0.442	1.557	0.316
Hx of Carotid Disease	1.407	0.625	4.084	0.024
Intercept	−0.952	0.711	0.386	0.180

^a^ Altered Mental Status was assessed based on level of consciousness (LOC), LOC Questions, and LOC Commands as defined in the National Institutes of Health Stroke Scale (NIHSS)

Table 3 below shows the confusion matrix associated with each of the three categories that are possible for the discharge diagnosis predicted variable.

**Table 3:** Confusion matrix for synthetic test set – RFE feature selection

	Predicted			Total
	TIA Mimics	TIA	Minor Stroke	
**Actual**				
**TIA Mimics**	19	6	1	**26**
**TIA**	8	43	1	**52**
**Minor Stroke**	1	3	8	**12**
**Total**	**28**	**52**	**10**	**90**

**Fig. 1:**
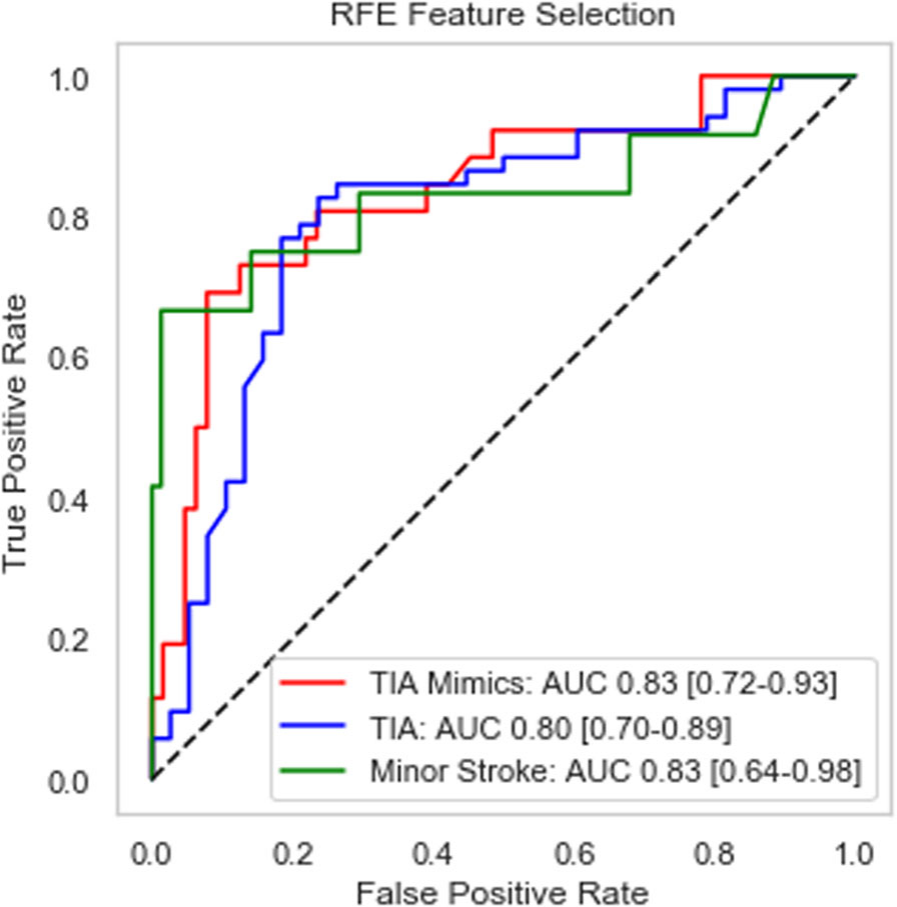
ROC curve and AUC measure for synthetic test set (*n* = 90)

Overall, the RFE-based classifier correctly predicts 78% of the overall observations (70 correct out of 90 total observations in the test set). In particular, the classifier correctly identifies 68% of the cases that resulted in a “TIA mimics” final diagnosis (19 out of 28), correctly identifies 83% of the “TIA” discharge diagnosis (43 out of 52), and correctly classifies 82% of the “minor stroke” discharge diagnosis (8 cases out of 10). A second measure used to evaluate the performance of a classifier is the Area Under the Curve (AUC), where the curve is the well-known Receiver Operating Characteristic (ROC). Figure 1 below shows the three ROC curves, with each AUC region hovering near 0.8, out of a theoretical maximum value of 1.0. Each AUC value is accompanied by a 95% confidence interval estimated by bootstrapping the test set.

We also evaluated the performance of the RFE-based classifier on the original data, that is, the original cohort of *n* = 269 cases. Recall that the original data set has a low prevalence of patients diagnosed with minor strokes and in our training phase we have used the SMOTE method to severely hybridize this set by over- and under-sampling. Thus, the original data set with all artificial data points removed is a better secondary benchmark, as opposed to a small validation subset which may suffer from little or no cases belonging to the “TIA” or “minor stroke” classes, respectively. Table 4 presents the results of the confusion matrix associated with the original cohort of 269 patients.

**Table 4:** Confusion matrix for original data set – RFE feature selection

	Predicted			Total
	TIA Mimics	TIA	Minor Stroke	
**Actual**				
**TIA Mimics**	48	52	3	**103**
**TIA**	21	110	4	**135**
**Minor Stroke**	4	18	9	**31**
**Total**	**73**	**180**	**16**	**269**

Consider that a naïve classifier would assign categories in accordance with the prior distributions of each class, that is, about 38% of data would be assigned to class 0, half the observations would be assigned class 1, and the remaining 12% would be assigned to class 2. The multinomial classifier outperforms the naïve across all categories. Specifically, we are able to correctly identify 66% of the “TIA mimics” discharge diagnostics (48 out of 73), 61% of the “TIA” diagnostics (110 out of 180), and 56% of the “minor stroke” category (9 cases out of 16). The overall accuracy of the classifier on the entire data set is 62% (167 cases correctly classified, out of 269 patients).

**Fig. 2:**
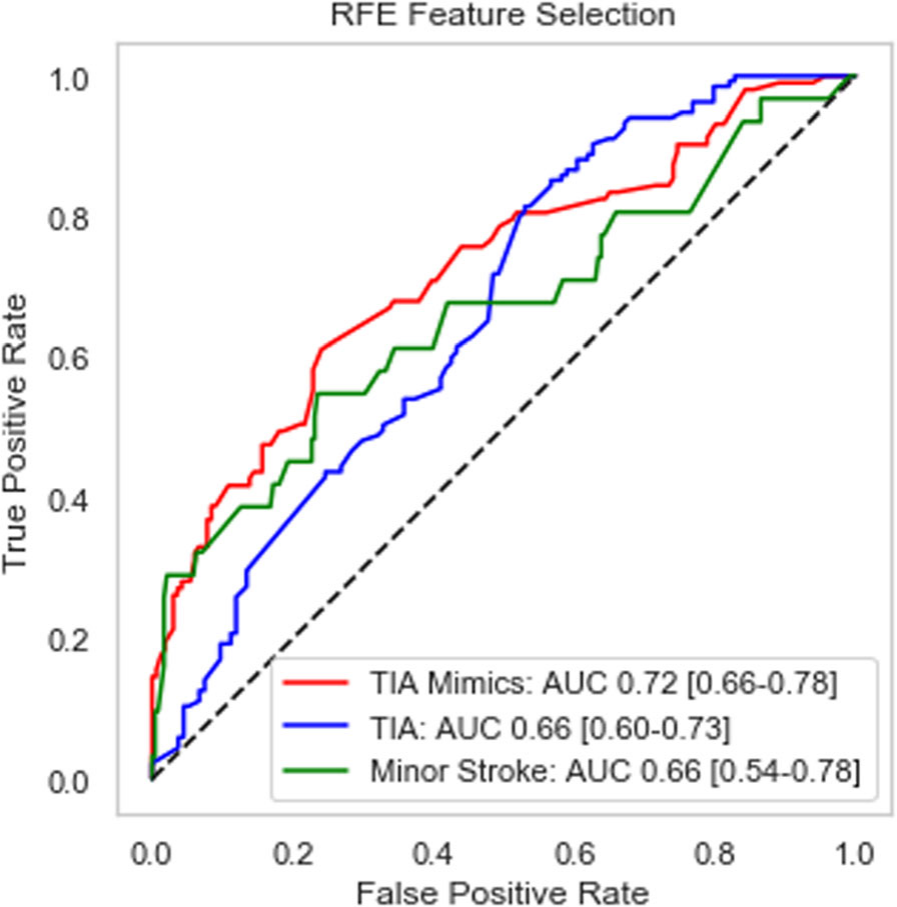
ROC curve and AUC measures for original data set (*n* = 269)

Like in the analysis for the synthetic test set, we provide an alternative way of evaluating the performance, via the ROC curves and the associated AUC measures, as depicted in Fig. 2 below. Each category exhibits a good lift, implying that our proposed classifier significantly outperforms random guessing, with each of the AUC measures hovering near 0.7.

We next evaluate the performance of the multinomial classifier based on the LASSO feature selection. In order to identify the best value for the penalty parameter *λ*, we performed a local grid search and found that the best performance happens when *λ* = 3.6 and approximately 30 features are retained. The LASSO-based classifier exhibits strong performance in identifying the three classes, as shown in Table 5 below. With respect to the augmented test set, the LASSO classifier has an overall accuracy of 74% (67 correct out of 90 total observations). The model is able to correctly identify 61% of the “TIA mimics” cases (16 out of 26 predictions), 79% of the “TIA” discharge diagnosis (41 out of 52) and 83% of the “minor stroke” diagnosis (10 cases out of 12). Note that in comparison to the performance of the RFE classifier on the synthetic test set (Table 3), the LASSO outperforms RFE with respect to the “minor stroke” class; however, it just slightly underperforms RFE with respect to the other two classes.

**Table 5:** Confusion matrix for synthetic test set – LASSO feature selection

	Predicted			Total
	TIA Mimics	TIA	Minor Stroke	
**Actual**				
**TIA Mimics**	16	10	0	**26**
**TIA**	9	41	2	**52**
**Minor Stroke**	1	1	10	**12**
**Total**	**26**	**52**	**12**	**90**

**Fig. 3:**
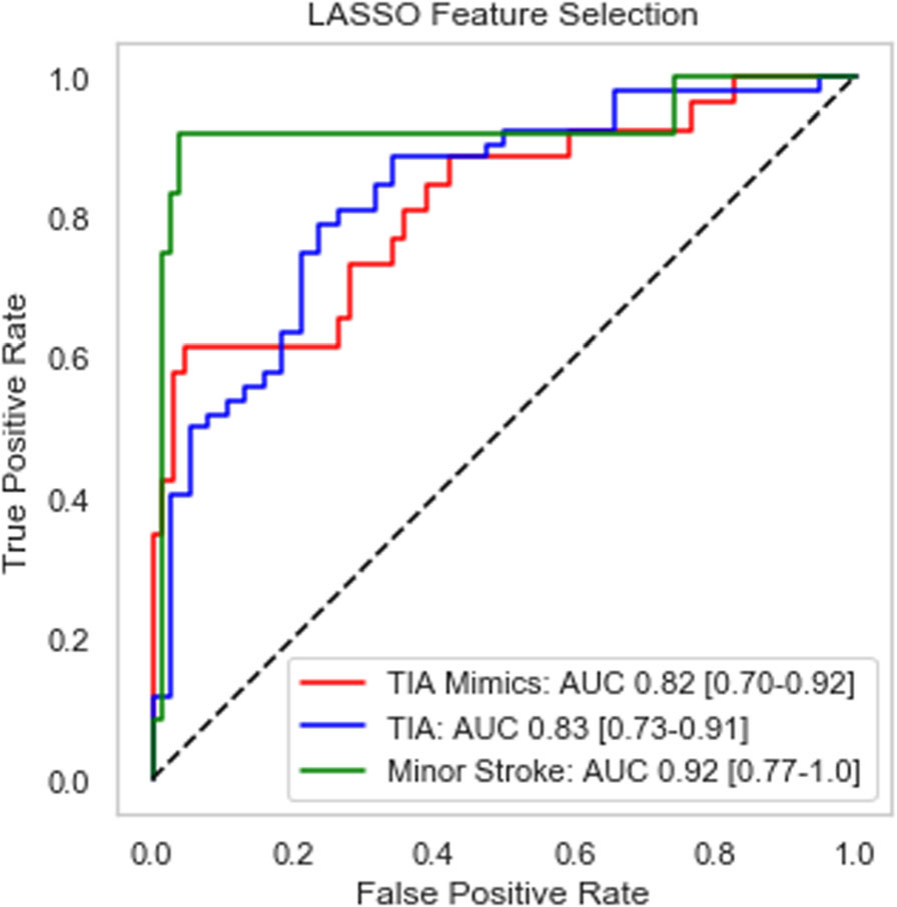
ROC curve and AUC measure for synthetic test set (*n* = 90)

The AUC performance on the synthetic test set is comparable to the RFE classifier and presented below in Fig. 3.

**Fig. 4:**
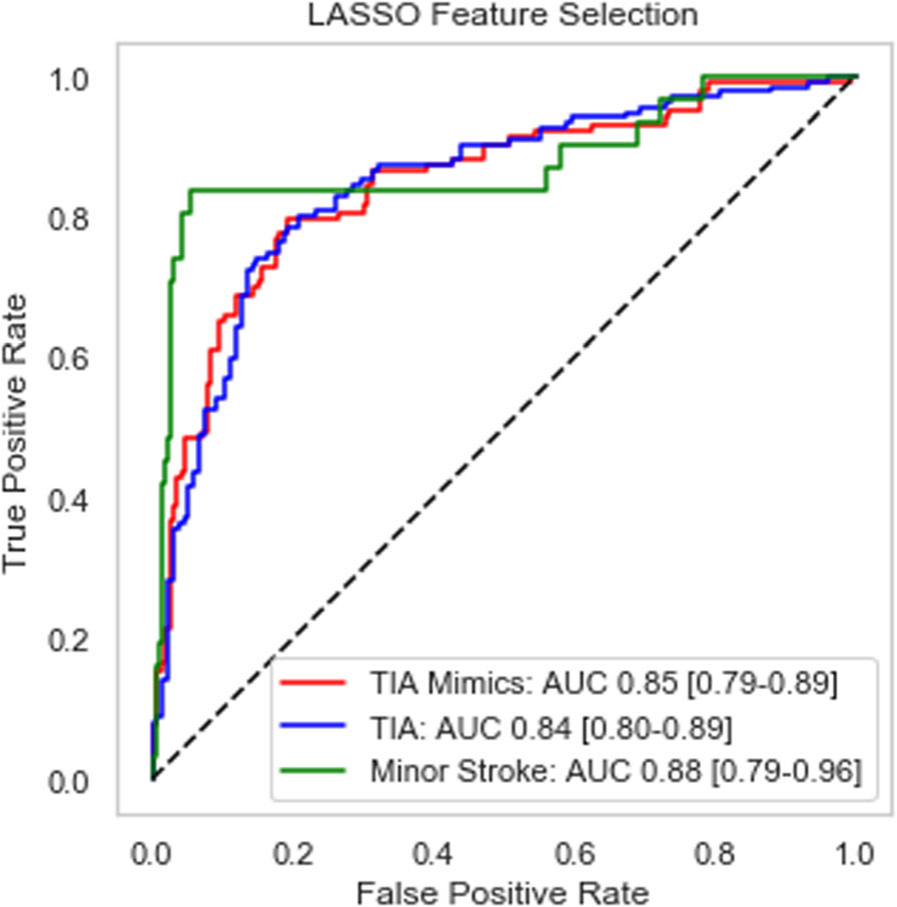
ROC curve and AUC measures for original data set (*n* = 269)

With regards to performance on the original data, the LASSO feature selection improves the prediction accuracy for all three outcome classes. In the former “TIA mimics” case, the LASSO-based model correctly predicts about 72% of the cases (76 correct predictions out of 105), while in the “minor stroke” category, the accuracy rate is about 68% (25 correct predictions out of 37). Finally, the “TIA” class prediction exhibits an accuracy of approximately 80% (101 correct predictions out of 127 cases). Similarly, the ROC curves show rapid growth for small false-positive rates, which indicate the ability of the LASSO-based classifier to discriminate correctly between the classes. Table 6 and Fig. 4 below summarize the confusion matrix and AUC values, respectively, for the LASSO classifier.

**Table 6:** Confusion matrix for original data set – LASSO feature selection

	Predicted			Total
	TIA Mimic	TIA	Minor Stroke	
**Actual**				
**TIA Mimic**	76	23	4	**103**
**TIA**	26	101	8	**135**
**Minor Stroke**	3	3	25	**31**
**Total**	**105**	**127**	**37**	**269**

Another way to evaluate the performance of the proposed classifiers is to compare them to established scoring methods from the existing clinical literature. For example, a well-known metric used in the ED for predicting the likelihood of a stroke following a TIA incident is the ABCD/ABCD2 score, which assigns to each patient a score from 0 to 7, based on four clinical features: Age, Blood Pressure, Clinical Features (such as unilateral weakness or speech disturbance) and the duration of symptoms (in minutes). Patients with scores of 6–7 are classified at high risk of experiencing a stroke within 7 days after a diagnose of TIA, patients with a score of 4–5 are classified as medium risk, with the remaining scores between 0 and 3 yielding a classification of low risk of stroke. There are some studies that indicated that higher ABCD2 scores may predict the diagnosis of a minor stroke, which may contribute to its predictive usefulness [5, 25]. Interestingly, no patients in the data set with an ABCD2 score of 6 or 7 ended up with a discharge diagnosis of stroke (that is, the accuracy of the ABCD2 “high risk of stroke given TIA” classification is 0%), so we opted to look at the accuracy of ABCD2 when both “medium risk” and “high risk” categories are merged to form a prediction for the stroke class. In a similar fashion, we can compare our two proposed classifiers to the Diagnosis of TIA (DOT) score, which is another scoring system well-established in the literature [26]. Table 7 below shows the performance of both the ABCD2 and DOT scores on the original dataset, relative to the proposed classifiers.

**Table 7:** Accuracy comparisons between ABCD2, DOT, and proposed classifiers

Discharge Diagnosis	ABCD2		DOT		Proposed Logistic Classifiers	
	Score	Accuracy (correct/total)	Score	Accuracy (correct/total)	RFE: Accuracy (correct/total)	LASSO: Accuracy (correct/total)
TIA Mimics	0–3	50.7% (68/134)	< −0.547	47.2% (17/36)	65.7% (48/73)	72.4% (76/105)
Minor Stroke	4–7	19.2% (26/135)	> −0.547	63.1% (147/233)	56.2% (9/16)	67.6% (25/37)
TIA					61.1% (110/180)	79.5% (101/127)
Aggregate	34.9% (94/269)		61.0% (164/269)		62.1% (167/269)	75.1% (202/269)

## Discussion

The results of this pilot study indicate that a multinomial classification model, based on a combination of feature selection mechanisms coupled with logistic regression, can be used to differentiate between TIA, TIA mimics, and minor stroke. We have also shown that our classifiers can make a more accurate diagnosis than DOT and ABCD2. While established methodologies such as the logistic regression have a more robust presence in the current literature and practice, in this study we explored the utilization of a multinomial logit model to facilitate distinguishing between three distinct outcomes.

There are not many well-validated tools for the diagnosis of TIA. Dawson score [27] and the DOT score [26] are two diagnostic scores that were developed based on regression analysis. A set of Explicit Diagnostic Criteria for TIA (EDCT) [28] for differentiating between migraine and TIA has also been recently proposed. Although these scoring systems have not been adequately validated and not been established as a useful tool in clinical practice, the DOT score was shown to perform better in a direct comparison with the Dawson score in a cohort of 525 suspected TIA patients (c-statistic 0.89 [0.85–0.92] versus 0.83 [0.79–0.87]) [26]. However, this comparison was performed in an internal validation of the DOT score.

Although there are few publications and no widely accepted definition for TIA mimics [29, 30], our study and other reports suggest that more than 50% of patients who are referred to TIA clinics are in fact TIA mimics [6, 7, 31]. Given a high estimated incidence rate of TIA in the United States, a high rate of misdiagnosis can be associated with significant cost burden and missed opportunities [32–34]. Misdiagnosing patients that are experiencing TIA carries significant costs for both the hospital and the patient [6]. These costs can be attributed to both patients and hospitals, and therefore developing an automated clinical decision support system to aid in the diagnosis of TIA is especially valuable. The prediction models developed in this study are performing better than the current tools and scoring systems such as ABCD2 and DOT and could be more effective when combined with other stroke risk stratification tools.

Our study has several limitations. Due to the combined retrospective and prospective nature of our study and limited sample size, we cannot implement this system in a clinical setting without fully validating these predictions prospectively and using a larger cohort from multiple health care systems. Also, in general, the problem at hand becomes more complicated due to the relatively large imbalance in the data among the three classes, coupled with a small TIA and stroke prevalence in the population [35, 36]. We addressed this limitation in part by implementing a data augmentation strategy based on the SMOTE algorithm, which improved the model performance; the downside of applying an algorithm such as SMOTE is the unnecessary amount of noise introduced in the training as set, as well as potential collinearity issues which may still result in a degradation in performance. Finally, the majority of patients in this study were Caucasian. This should be taken into the consideration when generalizing the results of this study. As a future direction, we plan on further enhancing our data augmentation strategy by using advanced machine learning algorithms such as Generative Adversarial Network (GAN). This will be important for practical applications of the system, such as improving the detection of rare conditions causing stroke mimics.

This study also has several key strengths that are rooted in the nature and source of the data, as well as in the study design considerations. One key strength is the use of only electronic health record (EHR) data. Using EHR data for building predictive models makes the integration of this automated system into a decision support system feasible, and without disruption of clinical workflow. In addition, the quality of our data is high, as it is validated by our neurologists. High-quality data translates into high-quality prediction models. However, we still understand that providers are likely to be biased in the information they input into the EHR system. To account for this shortcoming, in our study we used different note types (ED provider, neurology consultation, history and physical, and discharge summary notes) written by different providers.

## Conclusions

In this study we have developed two different multinomial models incorporating feature selection for differentiating between TIA and its mimics. The performance of these models seems promising. For other future directions, we intend to design quality control and noise filters to improve the quality of the EHR data to facilitate the implementation of a large-scale automated system for real-time use. Curated datasets, including this gold standard cohort of limited size, will play a key role in identifying and removing noise for large scale systematic data curation. Finally, our modeling framework is scalable and could be easily implemented as an automated tool in healthcare organizations to improve overall care and access, as well as to enhance secondary preventive measures and reduce diagnostic error at a personalized level. A carefully planned EHR-embedded decision support tool that could assist the ED providers in making an accurate diagnosis of TIA is an unmet need. This study suggests that it is feasible to build an automated decision support system using EHR data and advanced statistical tools. We also plan to further investigate if more complex machine learning models can be designed to enhance the performance, reliability, and accuracy of our models.

## Supplementary information

**Additional file 1: Supplementary Table 1.** Clinical and imaging elements considered for TIA diagnosis.

## Data Availability

The datasets generated for this study can be de-identified and made available by request to the corresponding author with appropriate data usage agreements.

## Abbreviations

ABCD2: Age/Blood Pressure/Clinical Features/Duration of Symptoms
AF: Atrial fibrillation
AUC: Area Under Curve
DOT: Diagnosis of TIA
ED: Emergency Department
EDCT: Explicit Diagnostic Criteria for TIA
GAN: Generative Adversarial Network
HTN: Hypertension
HER: Electronic health record
LASSO: Least Absolute Shrinkage and Selection Operator
LOC: Level of consciousness
NIHSS: National Institutes of Health Stroke Scale
RFE: Recursive Feature Elimination
ROC: Receiver Operating Characteristic
SMOTE: Synthetic Minority Over-sampling Technique
TIA: Transient ischemic attack
