# Supplementary material for: A predictive analytics model for differentiating between transient ischemic attacks (TIA) and its mimics
Source: BMC Med Inform Decis Mak. 2020 Jun 18;20:112. doi: 10.1186/s12911-020-01154-6 (PMC7302339; doi:10.1186/s12911-020-01154-6)
Supplement: Supplementary file 1 — Additional file 1: Supplementary Table 1. Clinical and imaging elements considered for TIA diagnosis. [file 12911_2020_1154_MOESM1_ESM.docx]

Supplementary Table 1. Clinical and imaging elements considered for TIA diagnosis

| **Feature** |
| --- |
| Age |
| Vascular risk factors (hypertension, elevated lipids, etc) |
| Onset (sudden/gradual/stuttering) |
| Duration of symptoms (minutes to hours) |
| Focal symptoms |
| Global symptoms (loss of consciousness, confusion) |
| Single versus multiple events (interval, last event) |
| Stereotyped versus variable |
| Vascular territory |
| Other medical history (seizure, migraine headache, atrial fibrillation etc.) |
| Associated symptoms |
| Brain MRI (previous ischemic lesion, microangiopathy, cerebral microbleeds) |
| Cerebral vascular imaging |
| Echocardiogram |
| Heart monitoring |
